# Supplementary material for: Climate change, not human population growth, correlates with Late Quaternary megafauna declines in North America
Source: Nat Commun. 2021 Feb 16;12:965. doi: 10.1038/s41467-021-21201-8 (PMC7886903; doi:10.1038/s41467-021-21201-8)
Supplement: Supplementary file 1 — Supplementary Information [file 41467_2021_21201_MOESM1_ESM.pdf]

SUPPLEMENTARY INFORMATION FOR "CLIMATE CHANGE, NOT HUMAN POPULATION GROWTH, CORRELATES WITH LATE QUATERNARY MEGAFAUNA DECLINES IN NORTH AMERICA"

Mathew Stewart
Extreme Events Research Group
Max Planck Institutes for
Chemical Ecology,
The Science of Human History, and
Biogeochemistry
Jena, Germany
mstewart@ice.mpg.de

W. Christopher Carleton
Extreme Events Research Group
Max Planck Institutes for
Chemical Ecology,
The Science of Human History, and
Biogeochemistry
Jena, Germany
wcarleton@ice.mpg.de

Huw Groucutt
Extreme Events Research Group
Max Planck Institutes for
Chemical Ecology,
The Science of Human History, and
Biogeochemistry
Jena, Germany
hgroucutt@ice.mpg.de

Contents

Supplementary Note 1: Radiocarbon-dated Event Count (REC) Modelling **3**

Supplementary Note 2: Chronologically Uncertain Covariates **5**

Supplementary Note 3: Regression Models **6**

Supplementary Note 4: Results 8

Supplementary Note 5: MCMC Diagnostics 8

Supplementary Note 6: Extended Analysis: Chronological uncertainty in the NGRIP record 10

Supplementary Note 7: Extended Analysis: Filtered megafauna data 14

Supplementary Note 8: Taking a recce of probable pasts: building and reading a Radiocarbon-dated Event Count Ensemble (RECE) 15

Supplementary Note 9: Additional Images 18

Supplementary Note 1: Radiocarbon-dated Event Count (REC) Modelling

Radiocarbon-dated Event Count (REC) models are a new approach designed to account for chronological uncertainty in records of radiocarbon-dated event times[1]. REC models involve sampling probable event count sequences in order to estimate one or more hyperparameters that reflect the uncertainty in individual event times. To begin, a Radiocarbon-dated Event Count Ensemble (RECE) is produced. A RECE is comprised of individual sequences, each constructed by randomly sampling a possible date for every event in a given radiocarbon date database. The event dates are sampled in accordance with the probability that the given event occurred on the relevant date. These probabilities are described by the relevant radiocarbon date density for the given event (usually a calibrated date density). Then, the number of events that fall into each interval of a sequence of intervals is counted thereby producing one probable event count sequence—one RECE member. Each member of the RECE is then used in an appropriately specified count-based regression model. These individual models can be thought of as a sample of regressions from a superpopulation of potential regressions involving all of the probable event count sequences. The parameters of these sample regressions are arranged in a multi-level Bayesian framework so that all of the relevant parameters and hyperparameter(s) can be estimated simultaneously. The hyperparameters characterize the superpopulation and, therefore, reflect the chronological uncertainty in individual event times.

Following[1], we used a Negative-Binomial (NB)-REC model. One of the key reasons for choosing this distribution is that it is appropriate for count data, but a more important reason is that it can account for *temporal spread*, which is a known effect of chronological uncertainty[2]. When we say we are uncertain about the date of a given event, we mean that we have some particular date for the event in mind and the probability that the event occurred at another date diminishes forward and backward in time away from the central, most likely date. The specific functional form of the relevant probability distribution—i.e., the probability we assign to each specific date—can vary quite a lot, as in the difference between a uniform function where we assign equal probabilities to every date within a given (usually finite) range and a calibrated radiocarbon date density, which is smoothly varying and typically multimodal. Regardless of functional form, given a large sample of uncertain event dates, the structure of this uncertainty will lead to a predictable distortion of the true event count sequence. Many or most sampled probable sequences will occupy a longer span of time than the true sequence. This distortion occurs because some events will sit at the ends of the temporal interval of interest. Portions of the densities of those leading and tailing events will extend beyond the majority of the sample, leading to a tapering effect. These tapered regions reflect lower probabilities of event dates relative to other portions of the interval of interest, which ultimately means fewer events likely occurred at these times. Consequently, any samples of probable event count sequences will include lower counts for these leading and tailing portions of the interval. Importantly, though, some probable sequences will contain non-zero counts for regions of time over which the tapering occurs even if none of the events actually occurred at those times. As a result, sampled sequences will tend to cover a longer span of time than the true event count sequence would.

An NB distribution can account for temporal spread because one of its parameters can be used to adjust the mean level of the process distribution. Imagine we have a time series of counts, with the count at any given time denoted y_t. We can model each observation in the series with a NB distribution as follows,

$$y_t \sim NB(r, p), \tag{1.1}$$

where $NB(\cdot)$ refers to the distribution, r is often thought of as the number of failures in a set of Bernoulli trials (e.g., coin flips), and "p" is the probability of success in a given trial[3]. The mean of this distribution can be written as follows,

$$\mu = r\frac{1-p}{p}. \tag{1.2}$$

With this parameterization of the mean, r can be defined by a regression model while p can then be applied like a weight to the regression term. Since it is a probability, p needs to be between 0 and 1, inclusive, which means that it can be used to pull the regression term down toward zero. Importantly, p can be allowed to vary as a function of time. As a result, the effects of chronological spread can be included because the p term can be allowed to have a greater impact—pull the regression term down more—over the leading and trailing ends of a given RECE member. So, re-writing r as a regression function and allowing p to vary, the NB model can be redefined as,

$$y_t \sim NB(r, p_t), \tag{1.3}$$

$$r = e^{\boldsymbol{X_t \beta}}, \tag{1.4}$$

where $\boldsymbol{X_t}$ is a row vector containing covariates for y_t, $\boldsymbol{\beta}$ is a column vector of regression coefficients, and the log-link function (the regression appearing in the exponent of e) is used to relate the linear function of predictors to the NB-distribution.

To account for the chronological uncertainty in individual event times, though, the model needs to be extended as described in[1]. Recall that REC models are based on estimating parameters at two levels. The lower level includes parameters for a number of individual regressions, each involving a single probable event count sequence (RECE member) as the response variable. The upper level contains hyperparameters that characterize the superpopulation of possible regressions and they reflect the chronological uncertainty in the dates of individual radiocarbon samples. The extended model can be written as follows,

$$y_{t,j} \sim NB(r_j, p_{t,j}), \tag{1.5}$$

$$r_j = e^{\boldsymbol{X_t \beta_j}}, \tag{1.6}$$

$$p_{t,j} \sim U(1e^{-10}, 1), \tag{1.7}$$

$$\boldsymbol{\beta_j} \sim MN(\boldsymbol{B}, \boldsymbol{\sigma}), \tag{1.8}$$

$$B_n \sim N(0, 100), \tag{1.9}$$

$$\sigma_n \sim U(1e^{-10}, 10). \tag{1.10}$$

In the above equations, $y_{t,j}$ refers to the count at time t in RECE member j, $MN(\cdot)$ refers to a multivariate Normal distribution, and $U(\cdot)$ refers to a Uniform distribution. The bold characters refer to vectors: $\boldsymbol{B}$ refers to a vector of N hyper-regression-coefficients corresponding to N covariates (including an intercept), while $\boldsymbol{\sigma}$ refers to a vector of N sampling uncertainties (standard deviations)—standard font versions of the same characters with subscript n refers to arbitrary elements of the corresponding vectors. The $p_{t,j}$ variables and hyperparameters, $\boldsymbol{B}$ and $\boldsymbol{\sigma}$, have priors for their parameters as indicated. So, for example, the $p_{t,j}$'s are restricted to be in $(0, 1]$ because of the software we used. To keep the notation simple, we wrote these priors directly into the equations (eq. 1.8–1.10). The priors were chosen to be at most weakly informative, as has been shown to be best-practice for Bayesian analyses aimed at parameter estimation[4,5].

Supplementary Note 2: Chronologically Uncertain Covariates

For the present study, we extended the NB-REC model further to account for chronological uncertainty in the covariates. When originally proposed, the REC model framework focused on chronological uncertainty in the response variable[1]. But, in the context of the present study, it is clear that the covariates contain chronological uncertainty as well. The human population size proxy, for instance, is the same as the megafauna one, namely a radiocarbon-dated event count proxy. This means that we can straightforwardly adapt the model to include samples from the a RECE of the human data. To do so, we rewrite the regression function, eq. 1.6, so that the covariate term refers to one probable covariate sequence as follows,

$$r_j = e^{\boldsymbol{X_{t,k} \beta_j}}. \tag{2.1}$$

$$\tag{2.2}$$

In this equation, k refers to one of K probable covariate sequences. Imagine randomly selecting one probable sequence from a human RECE. That sequence is then placed in a regression model, paired with a single probable event count sequence from a megafauna RECE.

We can think of r_j, then, as one probable regression involving a random pairing of probable megafauna and human event count sequences. The intercept never changes, so every probable intercept sequence is the same—a sequence of 1's. The same logic extends to the taphonomic proxy and any other chronologically uncertain covariate. The term r_j represents one combination of probable sequences—one probable regression. As long as the variables involved are independently-drawn probable sequences, the logic of multi-level modelling is maintained. The individual combinations are "exchangeable"[4] and the top-level hyperparameters reflect variation in the coincident combinations of values that might have occurred in the past given our uncertainty about the time stamps associated with the individual observations in each sequence.

Supplementary Note 3: Regression Models

We used our extended NB-REC regression models to test for relationships between megafauna population size and two potential explanatory variables, namely human population size and climate change. Following previous research[6], we ran several parallel analyses. Each one involved three models: 1) one in which the megafauna data were compared to a radiocarbon-date database comprised of human or anthropogenic samples (i.e., a human population proxy); 2) one in which the megafauna data were compared to the NGRIP oxygen isotope record (i.e., a climate change proxy); and 3) one model in which megafauna data were compared to both the human population proxy and the climate change proxy. In one of these three-model analyses, we used the whole megafauna radiocarbon-date database as the response variable for the regression models. Then, for the other analyses, we subdivided the megafauna radiocarbon-date database into the same species groupings used by Broughton and Weitzal (2018). These species sub-samples were slotted in as the regression response variable for each of the three models in a given analysis. This meant that in addition to the analysis involving the whole megafauna radiocarbon-date database, we ran analyses that looked separately at equus, mammoth, mastodon, sloth, and sabertooth count data. We also ran a further two analyses based on Broughton and Weitzel's (2018) regional subdivisions. One involved samples related to mastodon and mammoth found in the Great Lakes region, while the other involved samples related to Sloth and Mammoth found in the US Southwest. In each of these regional analyses, a separate set of models was created for the relevant megafauna species. It is important to note that we also used the same temporal divisions as Broughton and Weitzel's (2018) and, therefore, our primary models were restricted to the period from 15,000 BP to 11,700 BP (though, see the extended climatic analysis below).

In all of our analyses we included a proxy for taphonomic effects. As explained by Surovell et al. (2009), natural processes of decay, erosion, and sedimentation—collectively, *taphonomic processes*—can be expected to bias count-based time series of archaeological or palaeoenvironmental samples in a predictable way. As time goes on, taphonomic processes result in the loss of older samples from the archaeological and palaeoenvironmental records (or, those samples

may still be intact but less likely to be recovered). We can, therefore, expect count-based time-series of these samples to contain more recent samples than older samples—i.e., the records may contain a trend toward higher counts in more recent periods than older periods purely as as a function of taphonomic processes. We needed to account for this bias to avoid confounding taphonimc trends with the potential effects of human population size and/or climate change. So, we used a proxy for taphonomic processes recommended by Surovell et al. (2009) as a control variable in all of our regression models. This proxy is based on a northern hemispheric tephra record and can be interpreted as a regional indicator of the loss of evidence over time caused by taphonomy. By including this control variable, our models would be able to isolate the relevant effects from taphonomy. As with the other records, the taphonomic record contained chronological uncertainty. So, we followed the same procedure for including it in the regression models. We sampled probable tephra event count sequences, including each sampled sequence in one of the probable regressions as a covariate along with a probable human event count record and/or the NGRIP climate change proxy.

To determine whether megafauna population declines were likely related to human population increase, climate change, or both, we examined the posterior mean densities of the regression coefficients in the NB-REC models. We reasoned that if human population size, climate change, or both were important drivers of Late Quaternary megafauna extinction in North America, then the regression coefficient(s) associated with the relevant variable(s) would be significantly different from zero. More specifically, a given variable would be considered important if its corresponding posterior density estimate did not include zero within its 95% credible region. Since we included the taphonomic proxy in all models, any significant effects could be viewed as indications that a given variable was important even after accounting for trends in megafauna population levels caused by taphonomic processes.

The NB-REC regression models employed for this analysis involved an enormous number of parameters. In each model, there was a hyperparameter mean and dispersion parameter for every covariate's regression coefficient and an intercept, which meant there were 6 parameters for the models involving only one covariate and the taphonomic proxy and 6 for the models involving two covariates and the taphonomic proxy. There were also lower-level parameters that included regression coefficients for the intercept, covariate(s), and taphonomic proxy in each of the individual RECE member regressions—so, either 3 or 4 lower-level regression parameters depending on whether a given model involved one or two covariates, respectively. Lastly, there was a p parameter for every observation in every RECE member sequence that also had to be estimated, which meant an additional $T \cdot J$ parameters, where T is the length of a given sequence and J is the number of RECE members included in the model. At an annual resolution, then, a model involving only two covariates (plus an intercept and taphonomy proxy) spanning the 5000-year period of interest in the present study would require the estimation of 5009 parameters for a single RECE member and 250,009 parameters for a model involving only 50 RECE members.

With such an enormous number of model parameters to estimate, computational resources were a limiting factor. Thus, we decided to sub-sample the data to make computation feasible. We employed two sub-sampling strategies. First, we used RECE samples comprised of 50 probable event count sequences and, second, we sub-sampled the RECEs with respect to time so that only every 10th observation was included. The former, of course, means that we cannot fully

explore the tails of the target parameter value densities because we have not likely included enough of the variation in probable event count sequences to fully capture the chronological uncertainty in the relevant records. That said, some accounting of that uncertainty is, in our view, better than none. The second sub-sampling strategy meant that we examined every 10th year from the beginning of a given RECE's temporal span to the end of its span. Previous simulation work investigating REC models has shown that this sub-sampling has no obvious effect[1], but it should be noted that it clearly would have an impact if the process of interest had important high-frequency variation. It would also be a problem if the sub-sampling was too aggressive, involving inter-observation gaps so large that important patterns in the records were obscured. In the case of the megafaunal extinction question, however, even decadal sampling (as was done here) is a very high resolution relative to the millennial scale processes under investigation.

All analyses were conducted in R[8] using the Nimble package[9] for estimating Bayesian model parameters with MCMC. We then used "ggplot2"[10] and "ggpubr"[11] for plotting results and diagnostics. The R code can be found as a supplementary document provided with this paper and at `https://github.com/wccarleton/megafauna-na`.

Supplementary Note 4: Results

The core results are presented in the main paper. Figures 1–3 below display the results of our regression models described in the main paper, but not presented visually.

Supplementary Note 5: MCMC Diagnostics

As mentioned, the model parameters under investigation in this study were estimated with MCMC methods. This means it was important to determine whether the algorithms had produced unbiased, stable estimates of those parameters. MCMC algorithms proceed by iteratively selecting at random a set of values for a given model's parameters. Each iteration, the samples are recorded forming sequences called "chains", with one chain for each parameter. The chain for a given parameter needs to be stationary—i.e., have statistical properties that are the same for the whole sequence and any reasonably large sub-section of it. The chain also needs to contain enough samples to produce an unbiased estimate of the target distribution. In our analyses, we ran the MCMC algorithms for 2,000,000 iterations, meaning that we captured chains that contained 2,000,000 samples for each parameter in a given model.

To determine whether the chains were stationary and unbiased, we used a combination of visual inspection and a standard test based on the Geweke statistic[12]. Visually, stable chains should contain no obvious trends or patterns. When plotted, they should ideally look like a dense random sample resembling a thick line. The Geweke diagnostic indicates whether the chains have means that deviate significantly from stationarity. It works by comparing the mean and variance of the first portion of the chain to the last portion with a simple two-sample test for equality of means. If the chain was drifting—the long-run mean was changing throughout the simulation—then the Geweke test would indicate as much because the distribution of values at the beginning of the chain would be significantly different from those at the end. To perform the test, we used the "Geweke.diag" function in the "Coda" R package[13]. The function

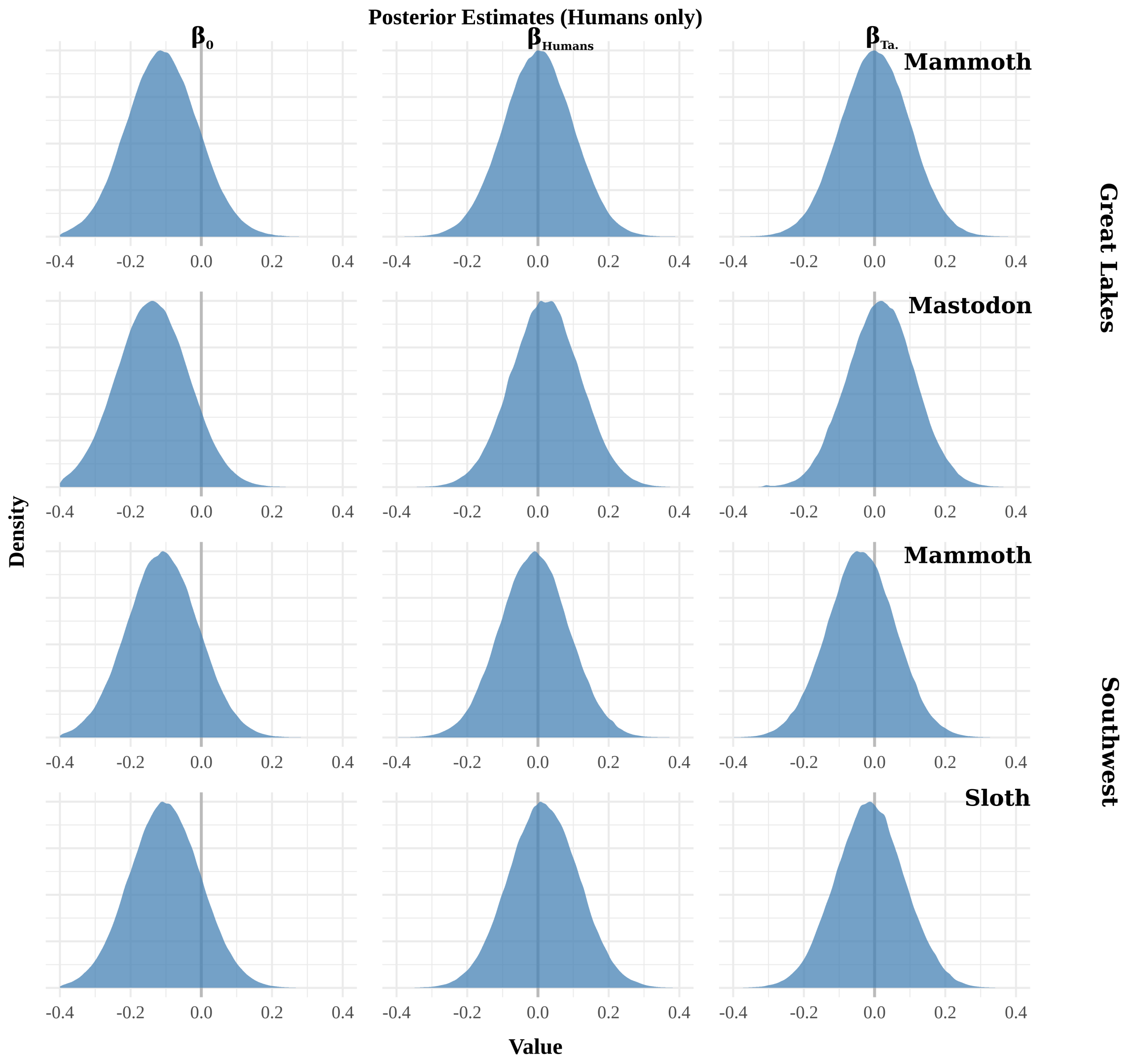

Supplementary Figure 1: Posterior densities for model parameter estimates from the regional analyses involving only the human demographic proxy data as the focal covariate.

produces a standard score (z-value) for each chain. Any chains with a standard score greater than ± 1.96 were inspected further (visually) for evidence of deviation from convergence. Given the large number of parameters in any given model, though, many Geweke diagnostic false alarms were to be expected. Sometimes, if a chain failed the Geweke test, a larger leading section of the chain would need to be discarded as burn-in before the remainder of the chain would pass the test and could, therefore, be used to estimate the target parameter's sampling distribution.

All of the chains in our analyses converged according to these diagnostics. We were, therefore, able to capture stable, unbiased estimates of the relevant model parameters. In each case, 20,000 of the leading iterations were discarded as burn-in prior to a given parameter's posterior distribution being plotted or analyzed further. However, as mentioned, each model involved thousands of parameters. So, we have only provided here images of MCMC chains for the main parameters in each model (see Figs. 4–6).

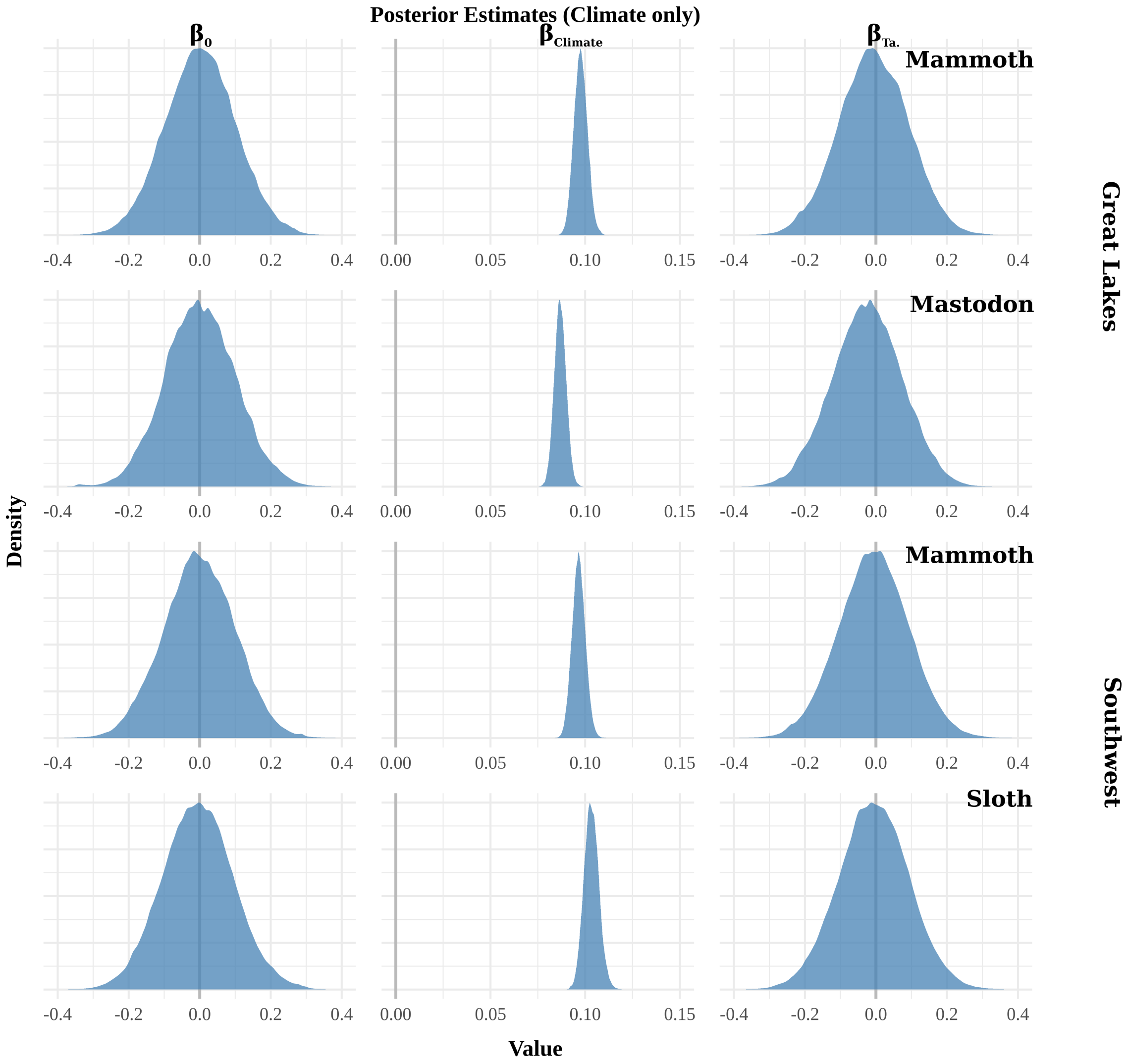

Supplementary Figure 2: Posterior densities for model parameter estimates from the regional analyses involving only the NGRIP (50-year running mean) climate proxy data as the focal covariate.

Supplementary Note 6: Extended Analysis: Chronological uncertainty in the NGRIP record

As Boers et al. (2017) explain, layer counted records can contain substantial chronological uncertainty. The presence of the uncertainty is somewhat surprising since these records are usually dated by counting visible layers in a structure that is created by a periodic lamination process of some sort—example structures include coral shells, tree rings, varved lakebeds, and of course ice cores. Counting errors, however, are ubiquitous in practice and can be caused by uncertainty in the visual, physical, or chemical distinctions between layers. This uncertainty is cumulative, which means that uncertainty about the date of any given layer in the record increases with the length of the record. Boers et al. (2017) found, for example, that the uncertainty in NGRIP layer-counted ice core records was as high as ± 2601 years by 60,000 years before the 2000 CE.

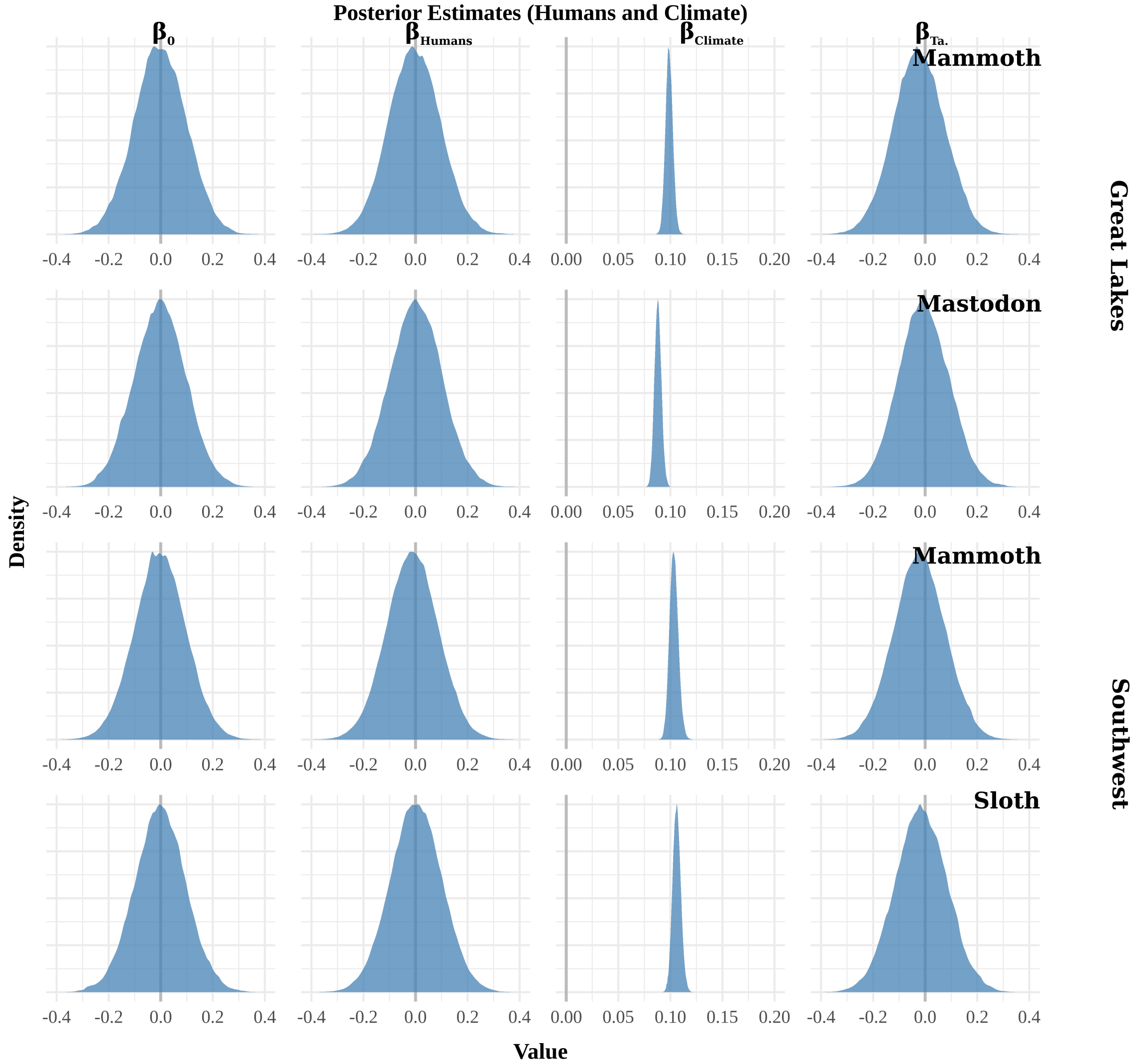

Supplementary Figure 3: Posterior densities for model parameter estimates from the regional analyses involving both the human demographic proxy data and the NGRIP (50-year running mean) climate proxy data as the focal covariates.

Boers et al. (2017) suggest accounting for this uncertainty by reprojecting it from the time-domain of the proxy onto its measurement domain. The process they developed amounts to determining the range of possible proxy values given both measurement error and dating uncertainty at a given time. It is a weighted average for a given time of all the measured values in a record where the weighting is determined by both the measurement error of each observation and, importantly, the estimated probability that each measurement dates to the time in question. The interpretation of the reprojection in the case of the NGRIP core, then, is the probable oxygen isotope ratio at a given time rather than the probable dates associated with a given measurement. Viewed this way, an uncertainty envelope can be produced around the NRGIP record. This means that we could sample probable NGRIP values for an arbitrary set of times and include that sample in a regression much like we had done with the other coviariates in our NB-REC models.

Using Boers et al.'s (2017) approach, therefore, we were able to account for chronological uncertainty in the NGRIP record. We first reprojected the NGRIP chronological uncertainty onto its measurement domain (see Supplementary

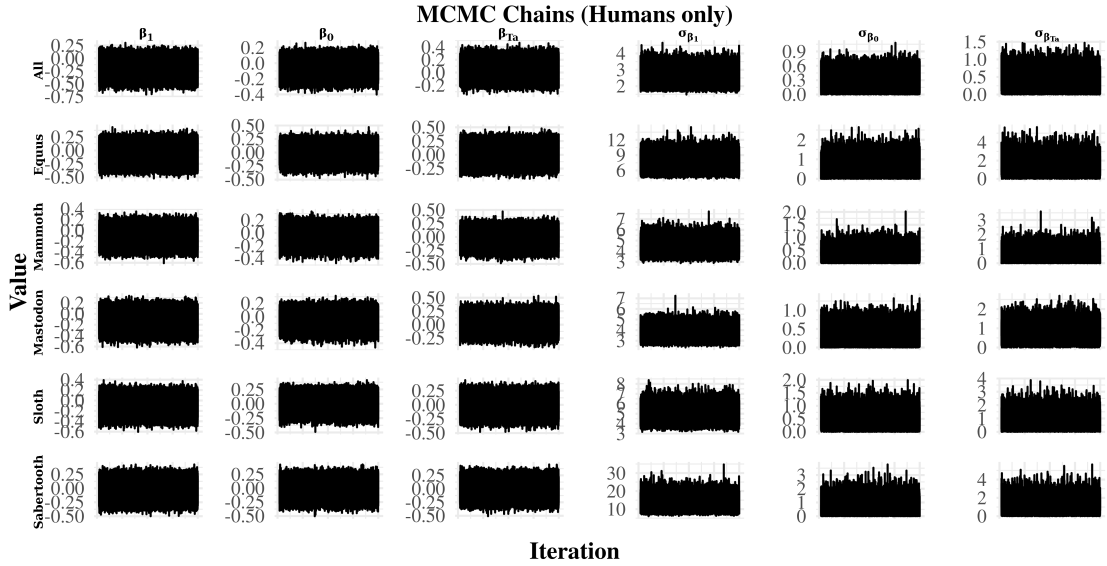

Supplementary Figure 4: MCMC chains for the models involving only the human population proxy as the relevant covariate.

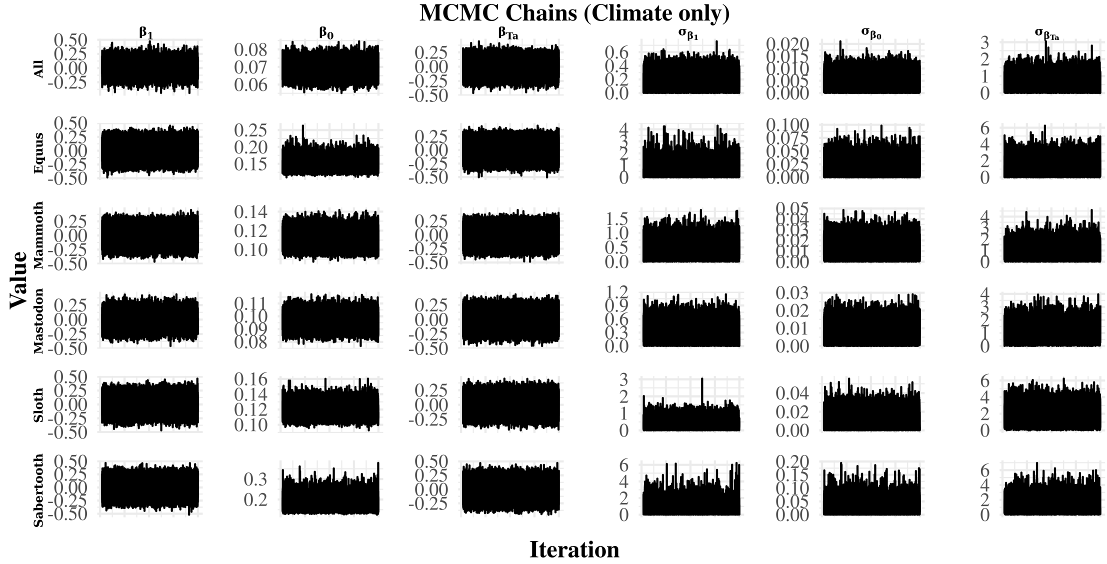

Supplementary Figure 5: MCMC chains for the models involving only the NGRIP 50-year smoothed oxygen isotope record (climate change proxy) as the relevant covariate.

Figure 7). Then, we created an ensemble of probable NGRIP sequences by sampling the newly adjusted measurement uncertainty envelope repeatedly in a point-wise fashion. These probable sequences were then included in a NB-REC regression model as covariates in the set of probable regressions alongside samples from the taphonomic proxy. Our objective was to test whether the non-zero NGRIP regression coefficient we observed in our primary analyses remained unchanged after accounting for layer-counted chronological uncertainty—i.e., we tested whether the posterior mean for the coefficient was positive and non-zero within its 95% credible interval.

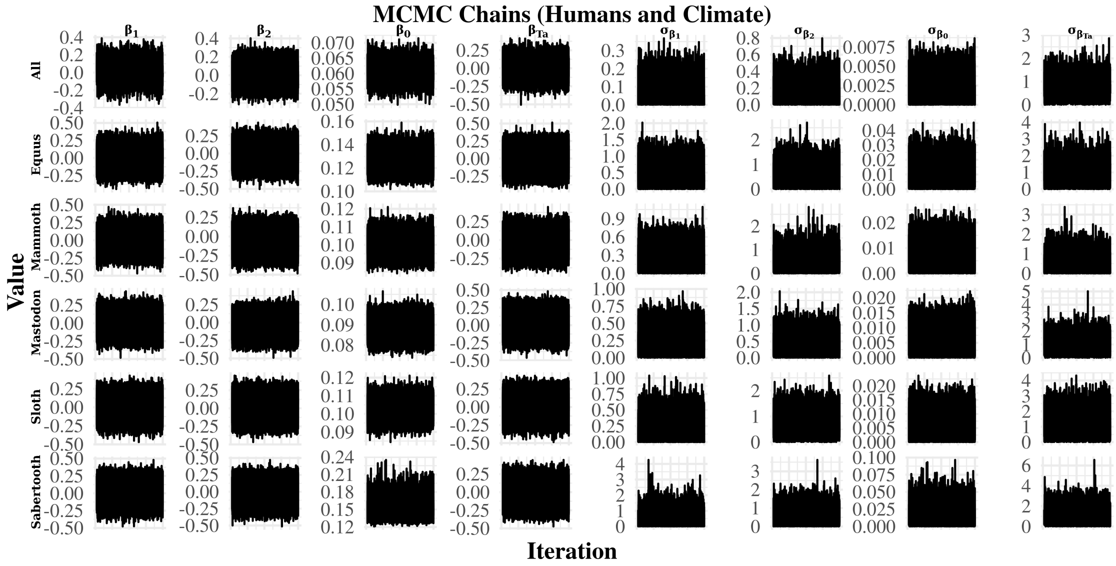

Supplementary Figure 6: MCMC chains for the models involving both the human population proxy and climate change proxy (NGRIP 50-year smoothed record) as the relevant covariate.

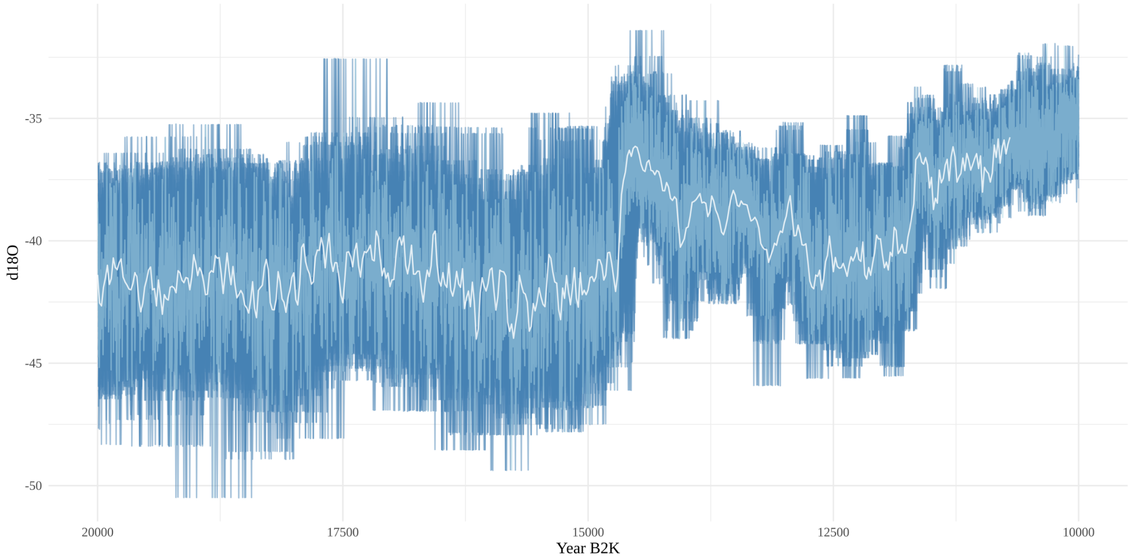

Supplementary Figure 7: Annual NGRIP oxygen isotope record with temporal uncertainty reprojected into the measurement domain. The white line represents the smoothed (50-year running mean) NGRIP record used in previous research and the main analyses of our study. The light blue line represents the raw annual record. The dark blue area represents the 95% uncertainty envelope created by reprojecting temporal uncertainty from the time domain (x-axis) onto the measurement domain (y-axis) using Boers et al.'s (2017) approach.

The findings of this extended analysis are consistent with our primary results. The posterior mean of the top-level regression coefficient for the climate proxy was positive in all cases and close to the previous values (see the main paper for the relevant figure). Thus, whether analyzing all megafauna species together, or separating the samples by species, there appears to be a fairly consistent positive relationship with the NGRIP proxy record. This estimate accounts for chronological uncertainty, and NGRIP measurement uncertainty. Importantly, it is also based on annually-resolved

data, which means that our primary findings cannot be dismissed as a result of using smoothed proxy data. It should be noted that the annual record is unsurprisingly more variable over short intervals than the smoothed record is. Thus, the consistency of our findings suggests that the relationship between the records is robust.

Supplementary Note 7: Extended Analysis: Filtered megafauna data

As we mentioned in the Methods section of the main paper, there is reason to think some overcounting may have occurred in the megafauna count series. Some of the megafauna radiocarbon samples in the database we analyzed are derived from contexts that have an unclear relationship to individuals. Many, for instance, are taken from dung or fragmented bone. Since a single animal could produce many such samples, those with date densities that overlap significantly could plausibly come from one animal. This would mean that individuals may have been overcounted in the final count-series. Given the small number of observations in the database to begin with, even a few over-counted individuals might skew the results of an analysis such as ours.

With this problem in mind, we created a new, filtered dataset and ran a supplementary set of regression models to see if overcounting may have affected our findings. First, we combed through the database and compared potentially overlapping samples from non-uniquely-identifiable sources (e.g., dung). Any samples with date densities that overlapped with any other densities were flagged—“overlapped” in this case meant the respective calibrated radiocarbon date densities were non-zero over some common interval. The samples corresponding to the overlapping densities were then further investigated to see if they could be distinguished on zooarchaeological or stratigraphic grounds based on the publications in which the samples were originally described/reported. Those samples that could not be separated were then removed to create a new aggressively filtered dataset. This had the effect of undercounting some samples, of course, but a trade off of some kind was inevitable.

Then, we ran a series of regression models as before. For this extended analysis, we began with the largest model, which included both the human population proxy and NGRIP climate change proxy as covariates. For the NGRIP covariate we used the annually-resolved record and sampled the reprojected ensemble again in order to account for chronological uncertainty in that time series following Boers et al. 2017. The taphonomic record was, of course, including again to control for large-scale long-term taphonomic biases.

As figure 8 shows, the results of this extended analysis were consistent with our previous findings. There were some small differences in estimated parameter values, as one would expect, but the overall pattern remained the same. Thus, even after aggressively filtering the megafauna data to reduce overcounting, there appears to have been no long-term relationship between the remaining megafauna sample counts and the human population indicator. There does appear, however, to be a consistent positive relationship between the NGRIP temperature proxy and megafauna sample counts. This pattern was the same whether megafauna taxa were pooled, or separated following recent research trends.

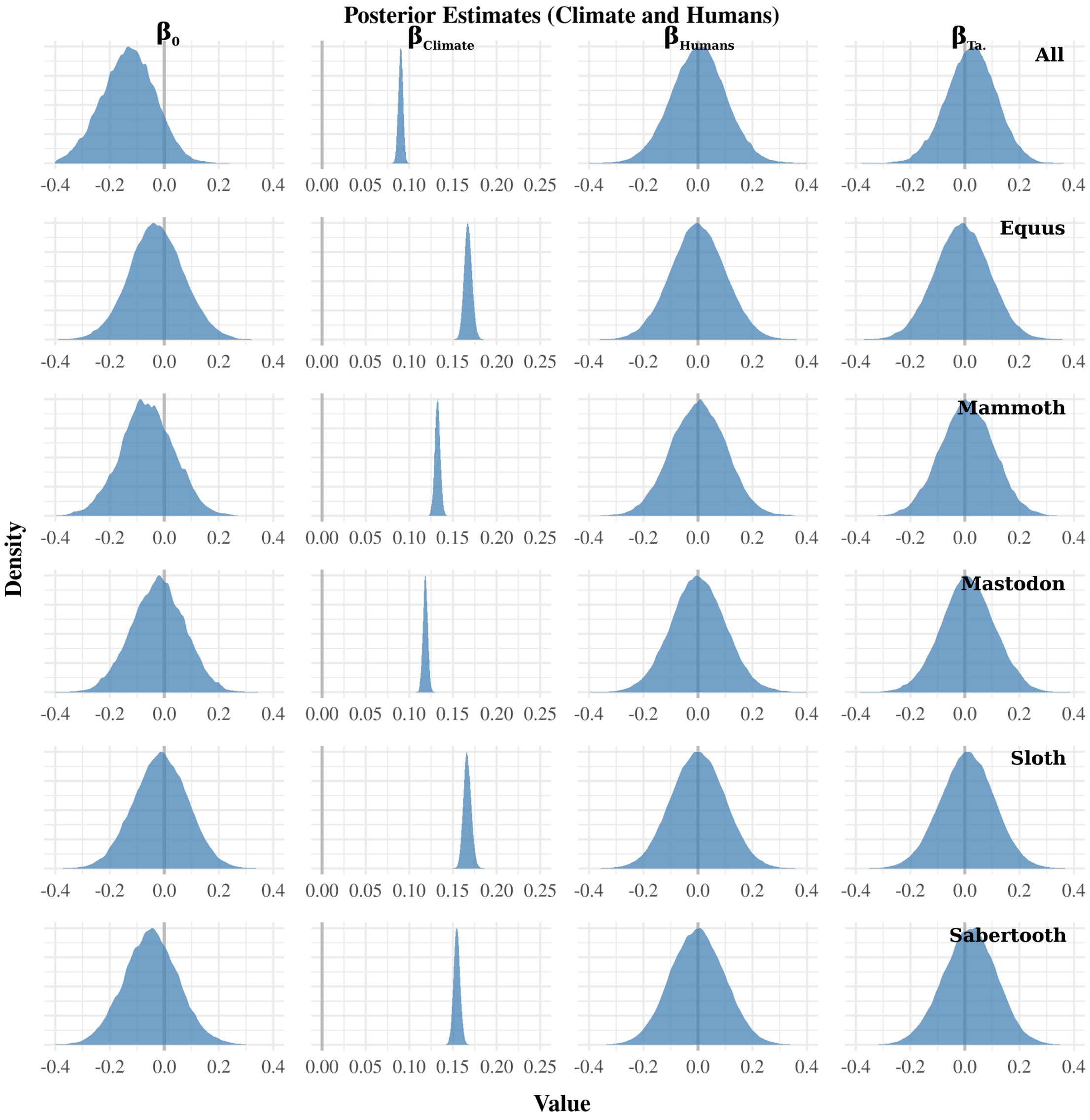

Supplementary Figure 8: Posterior densities for a series of REC models involving the filtered megafauna dataset.

Supplementary Note 8: Taking a recce of probable pasts: building and reading a Radiocarbon-dated Event Count Ensemble (RECE)

A Radiocarbon-dated Event Count Ensemble is a collection of probable event-count sequences compatible with the chronological uncertainties associated with the timing of the relevant events. To clarify, we can imagine creating a very simple RECE based on only two events. The events could represent anything dated with radiocarbon, but for simplicity imagine two separate, distinct hearths uncovered in an archaeological excavation. These imaginary hearths were each used only once and, so, represent spatially and temporally discrete activity "events" (a few hours of use each). The hearths contain seeds—short-lived carbon samples—that have been dated. Thus, our imaginary dataset contains two radiocarbon-dated events, each with a distribution of probable dates represented by the corresponding radiocarbon-date

probability density functions estimated from the two carbon samples. Strictly speaking, the radiocarbon-date probability functions are actually discrete estimates of underlying continuous density functions (reflecting, ultimately, isotope measurement uncertainty) and, so, the distributions typically returned by calibration software could properly be referred to as "probability mass functions". But the principles are all the same, so we will refer here simply to "probability distributions". For the time being we can also ignore radiocarbon-date calibration altogether—it ultimately only exacerbates the challenges of dealing with chronologically uncertain event times, but the core problems arise even without it.

To build the RECE, we need to randomly sample the two radiocarbon-date distributions and then turn those two dates into a count sequence. This entails randomly selecting a date for each of the two events from the relevant distributions in such a way that the probability of selecting a particular date corresponds to the the height of a given distribution—i.e., the higher the level of the distribution, the more likely the corresponding date will be drawn. Each time we sample the distributions in this way, we obtain two probable event dates. These dates can then be converted into a count sequence. The sequence is created by establishing a temporal grid and counting the number of events that fall into each interval in the grid. To continue keeping things simple, we can image the resolution of the grid is annual and, so, we are counting the number of events (hearths) that are dated to a given year. This time-series of counts constitutes a single probable event-count sequence (i.e., one RECE member). The same sampling and counting procedure needs to be repeated many times to build the ensemble.

Once built, the RECE is stored in a matrix. Each row in the matrix corresponds to a year, and each column contains a single probable count sequence. Each sequence is a random sample that represents a probable time series of event counts, one probable history of hearth counts. With one or two sequences, it would be trivially easy to visualize and interpret the RECE. Each probable sequence could simply be plotted separately in a standard time-series plot and viewed as alternate histories. But, each individual sequence is a highly biased estimate of the true event-count sequence. While one sequence of events actually occurred, our uncertainty about the timing of the events means we cannot be certain which of the sampled sequences best represents the true one. With even just two radiocarbon-date distributions to consider (e.g., only two hearth features to worry about counting) there are many probable sequences. So, typically, a RECE will need to be comprised of many sequences. Looking at each one in a separate plot would be tedious, but more importantly the exercise would fail to reveal an overall picture that could be readily interpreted. Instead, we need to collapse the enormous amount of information in a typical RECE into only two dimensions for plotting.

With helpful reviewer feedback, we have used a sort of heat-plot approach to plotting RECEs. To build the plot we first have to create an *agreement matrix* from the RECE. Like a RECE itself, the rows of the agreement matrix correspond to the years in the RECE's temporal grid, but the columns refer to the count of events rather than individual sequences. For each cell in the matrix, the number of RECE members that contain a given count for a given year are tallied up. The tallies in each cell indicate the level of agreement among RECE members about the event count for a given date. These cell values can then be used to create a heat-map, with higher tallies (greater agreement) indicated by hotter, brighter colours. An important exception occurs when no RECE members contain a given count for a given year. In that case,

the agreement matrix contains a tally of zero for the cell corresponding to the relevant year-count intersection. In the heat-map, such cells are represented by no colour at all, which means that transparent portions of the heat-map indicate agreement among RECE members that the true count sequence probably does not contain the relevant year-count pairing, at least given the sample of sequences in the RECE.

Take, for example, the human RECE in Supplementary Figure 7 of the main text. This RECE is built from the CARD database and effectively counts archaeological sites from 20,000–10,000 BP. In Supplementary Figure 9 we have zoomed in on a portion of this RECE plot. We have also drawn a line around a given year column in the plot (11704 BP) and written in the agreement matrix values for the relevant count levels in that column. The agreement matrix indicates that 171 RECE members have a count of 1 for the year 11704 BP; 20 others have a count of 2 for that year; 0 have a count of 3; 0 have a count of 4; and 1 has a count of 5.

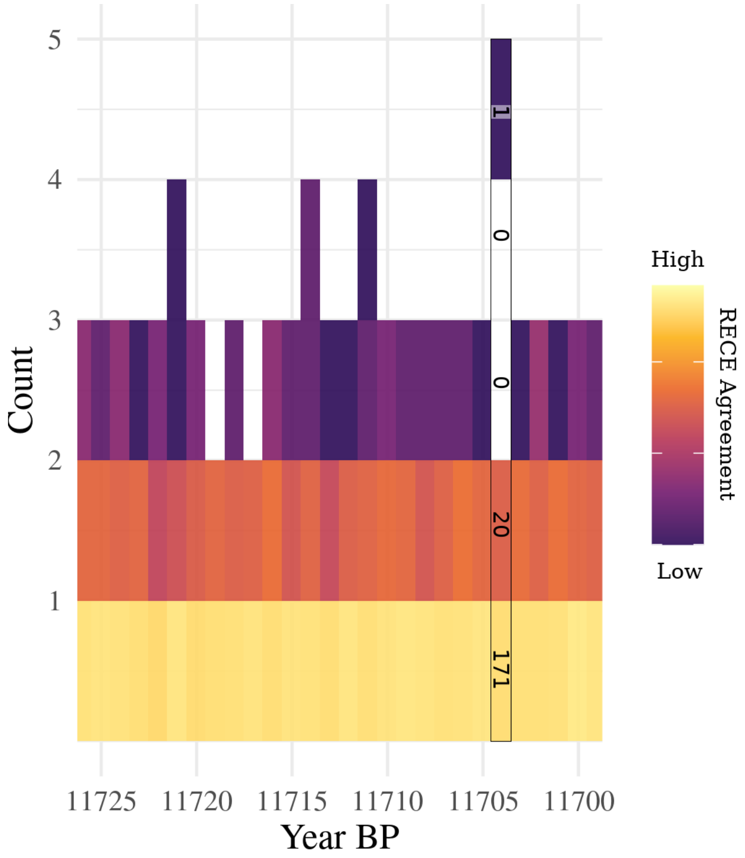

Supplementary Figure 9: Zoomed-in section of the Human RECE plot from Figure 7 of the main text. The numbers in the outlined column indicate the number of RECE members that have the corresponding count on the y-axis for the given year on the x-axis. Higher numbers indicate greater "agreement" among probable event-count sequences, except were the count is zero. In the case of zeros, there is also "agreement" in the sense that none of the sampled sequences contain the relevant count-year combination. The heat-map colour scale is plotted on the right of the figure and the numbers (agreement values) have been logarithmically stretched in order to enhance contrasts.

Supplementary Note 9: Additional Images

Additional images mentioned in the text are presented below.

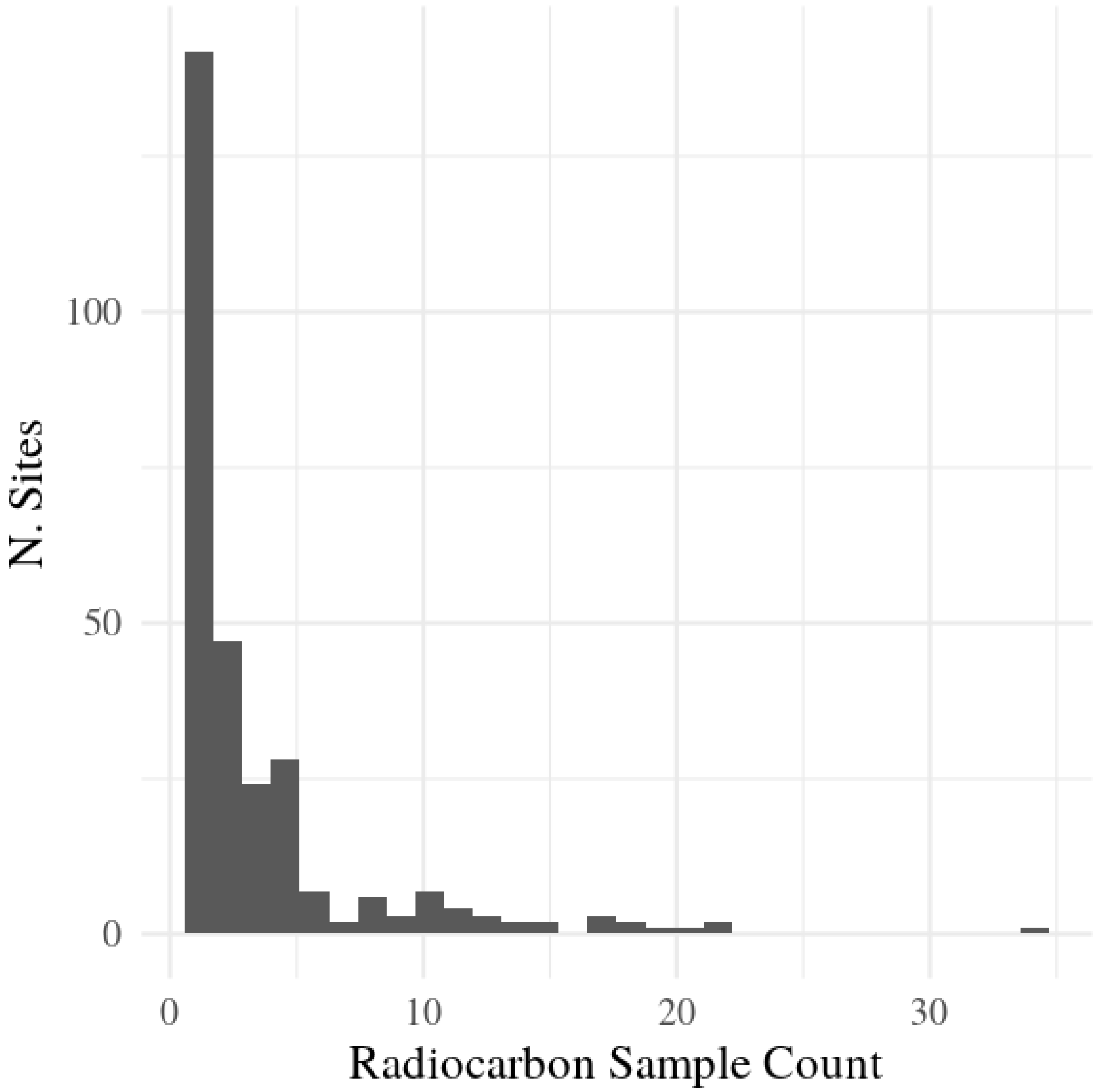

Supplementary Figure 10: Histogram showing the distribution of sites in the CARD database containing a given number of radiocarbon samples. As the plot indicates, the vast majority of sites are represented in the database by a fairly even, low number of samples. These archaeological sites date roughly to between 15,000 cal. BP and 11,700 cal. BP and the data have been cleaned as described in the Methods section of the main text.

References

[1] William Christopher Carleton. Evaluating Bayesian radiocarbon-dated event-count modelling for the study of long-term human and environmental processes. *Journal of Quaternary Science*, 36:110–133, 2020. doi: 10.1002/jqs.3256.

[2] Christopher Bronk Ramsey. Methods for Summarizing Radiocarbon Datasets. *Radiocarbon*, 59(6):1809–1833, 2017. doi: 10.1017/RDC.2017.108.

[3] Joseph M Hilbe. *Negative Binomial Regression*. 2011. ISBN 9780521198158.

[4] Andrew Gelman, John B Carlin, Hal S Stern, David B Dunson, Aki Vehtari, and Donald B Rubin. *Bayesian Data Analysis*. CRC Press, Boca Raton, 3rd edition, 2013. ISBN 978-1-4398-9820-8. doi: 10.1007/s13398-014-0173-7.2.

[5] Jonah Gabry, Daniel Simpson, Aki Vehtari, Michael Betancourt, and Andrew Gelman. Visualization in Bayesian workflow. *Journal of the Royal Statistical Society. Series A: Statistics in Society*, 182(2):389–402, 2019. doi: 10.1111/rssa.12378.

[6] Jack M. Broughton and Elic M. Weitzel. Population reconstructions for humans and megafauna suggest mixed causes for North American Pleistocene extinctions. *Nature Communications*, 9(1):1–12, 2018. ISSN 20411723. doi: 10.1038/s41467-018-07897-1.

[7] Todd A. Surovell, Judson Byrd Finley, Geoffrey M. Smith, P. Jeffrey Brantingham, and Robert Kelly. Correcting temporal frequency distributions for taphonomic bias. *Journal of Archaeological Science*, 36(8):1715–1724, 2009. doi: 10.1016/j.jas.2009.03.029.

[8] R Core Team. R: A Language and Environment for Statistical Computing, 2020. URL `https://www.r-project.org`.

[9] NIMBLE Development Team. Nimble user manual, 2018. R package manual version 0.6-12.

[10] Hadley Wickham. *ggplot2: Elegant Graphics for Data Analysis*. Springer-Verlag New York, 2016. ISBN 978-3-319-24277-4.

[11] Alboukadel Kassambara. *ggpubr: 'ggplot2' Based Publication Ready Plots*, 2019. R package version 0.2.2.

[12] John Geweke. Evaluating the Accuracy of Sampling-Based Approachesto the Calculation of Posterior Moments. In J.M. Bernardo, J.O. Berger, A.P. Dawid, and A.F.M. Smith, editors, *Bayesian Stastistics*, pages 169–193. Clarendon Press, Oxford, 4 edition, 1992.

[13] Martyn Plummer, Nicky Best, Kate Cowles, and Karen Vines. Coda: Convergence diagnosis and output analysis for mcmc. *R News*, 6(1):7–11, 2006. URL `https://journal.r-project.org/archive/`.

[14] Niklas Boers, Bedartha Goswami, and Michael Ghil. A complete representation of uncertainties in layer-counted paleoclimatic archives. *Climate of the Past*, 13(9):1169–1180, 2017. ISSN 1814-9332. doi: 10.5194/cp-13-1169-2017.
