# Supplementary material for: Climate change, not human population growth, correlates with Late Quaternary megafauna declines in North America
Source: Nat Commun. 2021 Feb 16;12:965. doi: 10.1038/s41467-021-21201-8 (PMC7886903; doi:10.1038/s41467-021-21201-8)
Supplement: Supplementary file 3 — Description of Additional Supplementary Files [file 41467_2021_21201_MOESM3_ESM.pdf]

Description of Additional Supplementary Files

File Name: Supplementary Data 1
Description: Radiocarbon-dated megafauna dataset (csv.).

File Name: Supplementary Data 2
Description: Radiocarbon-dated megafauna dataset (xlsx.) with megafauna removed during additional data cleaning flagged.

File Name: Supplementary Data 3
Description: North Greenland Ice Core Project (NGRIP) δ18O record.

File Name: Supplementary Data 4
Description: Calibrated radiocarbon database of late Quaternary volcanic eruptions from Bryson, R.U., Bryson, R.A., Ruter, A. [2006. A calibration radiocarbon database of late Quaternary volcanic eruptions. Earth Discussions 1, 123–124].

File Name: Supplementary Code 1
Description: R-markdown zip file required to replicate the study.
