# Supplementary material for: Climate change, not human population growth, correlates with Late Quaternary megafauna declines in North America
Source: Nat Commun. 2021 Feb 16;12:965. doi: 10.1038/s41467-021-21201-8 (PMC7886903; doi:10.1038/s41467-021-21201-8)
Supplement: Supplementary file 9 — Reporting Summary [file 41467_2021_21201_MOESM9_ESM.pdf]

nature research

Corresponding author(s): Mathew Stewart
W. Christopher Carleton
Huw S. Groucutt

Last updated by author(s): 13.01.2021

Reporting Summary

Nature Research wishes to improve the reproducibility of the work that we publish. This form provides structure for consistency and transparency in reporting. For further information on Nature Research policies, see our Editorial Policies and the Editorial Policy Checklist.

Statistics

For all statistical analyses, confirm that the following items are present in the figure legend, table legend, main text, or Methods section.

n/a	Confirmed	
☐	☒	The exact sample size (n) for each experimental group/condition, given as a discrete number and unit of measurement
☐	☒	A statement on whether measurements were taken from distinct samples or whether the same sample was measured repeatedly
☐	☒	The statistical test(s) used AND whether they are one- or two-sided<br>*Only common tests should be described solely by name; describe more complex techniques in the Methods section.*
☐	☒	A description of all covariates tested
☐	☒	A description of any assumptions or corrections, such as tests of normality and adjustment for multiple comparisons
☐	☒	A full description of the statistical parameters including central tendency (e.g. means) or other basic estimates (e.g. regression coefficient) AND variation (e.g. standard deviation) or associated estimates of uncertainty (e.g. confidence intervals)
☒	☐	For null hypothesis testing, the test statistic (e.g. F, t, r) with confidence intervals, effect sizes, degrees of freedom and P value noted<br>*Give P values as exact values whenever suitable.*
☐	☒	For Bayesian analysis, information on the choice of priors and Markov chain Monte Carlo settings
☐	☒	For hierarchical and complex designs, identification of the appropriate level for tests and full reporting of outcomes
☒	☐	Estimates of effect sizes (e.g. Cohen's d, Pearson's r), indicating how they were calculated

Our web collection on statistics for biologists contains articles on many of the points above.

Software and code

Policy information about availability of computer code

Data collection	Data were collected with R.4.03 (https://r-project.org)
Data analysis	All analyses were conducted in R (4.03) using a combination of established packages and custom code, and is provided with the published article (Supplementary Code). The following R packages were used in the analysis and figure production: nimble (0.9.1), ggplot2 (3.3.2), ggpubr (0.4.0), clam (2.3.5), tidyr (1.1.2), and abind (1.4.5).

For manuscripts utilizing custom algorithms or software that are central to the research but not yet described in published literature, software must be made available to editors and reviewers. We strongly encourage code deposition in a community repository (e.g. GitHub). See the Nature Research guidelines for submitting code & software for further information.

Data

Policy information about availability of data

All manuscripts must include a data availability statement. This statement should provide the following information, where applicable:

- Accession codes, unique identifiers, or web links for publicly available datasets
- A list of figures that have associated raw data
- A description of any restrictions on data availability

All data generated or analysed during this study are included in the published article (and its Supplementary Data files) with the exception of the human radiocarbon sample database, which can be provided on request or retrieved directly from CARD, but cannot be openly shared according to the policies of the source database (https://www.canadianarchaeology.ca/).

Field-specific reporting

Please select the one below that is the best fit for your research. If you are not sure, read the appropriate sections before making your selection.

☐ Life sciences ☐ Behavioural & social sciences ☒ Ecological, evolutionary & environmental sciences

For a reference copy of the document with all sections, see nature.com/documents/nr-reporting-summary-flat.pdf

Ecological, evolutionary & environmental sciences study design

All studies must disclose on these points even when the disclosure is negative.

Study description	Here we used a recently developed Bayesian regression technique—Bayesian Radiocarbon-dated Event Count (REC) Modelling—to test whether late Quaternary declines in North American megafauna could be best explained by human population growth, climate change, or both. The dependent variable in all cases was comprised of radiocarbon-dated events pertaining to megafauna fossils from North America. Two independent variables were used; one comprised archaeological radiocarbon-dated samples from North America; and the other was the North Greenland Ice Core Project (NGRIP) oxygen isotope record.
Research sample	The human radiocarbon sample was obtained from the CARD database (https://www.canadianarchaeology.ca/). The megafauna radiocarbon sample and North Greenland (NGRIP) oxygen isotope record were obtained from published sources.
Sampling strategy	All data used in the present study were obtained from pre-existing databases.
Data collection	All data used in the present study were obtained from pre-existing databases.
Timing and spatial scale	No field or experimental analyses were conducted to obtain the data. All data were collected from pre-existing databases.
Data exclusions	In our extended analysis we conducted additional "chronometric cleaning" by removing any instances where individual radiocarbon dates may have originated from the same megafauna individual. This cleaning resulted in the exclusion of 89 radiocarbon dates.
Reproducibility	All data and code required to replicate the analysis are provide with the paper, with the exception of the human radiocarbon sample database which can be obtained upon request from the authors or from the CARD (https://www.canadianarchaeology.ca/)
Randomization	All available human and megafauna dates were used in the analysis.
Blinding	No experiments were conducted for the present study

Did the study involve field work? ☐ Yes ☒ No

Reporting for specific materials, systems and methods

We require information from authors about some types of materials, experimental systems and methods used in many studies. Here, indicate whether each material, system or method listed is relevant to your study. If you are not sure if a list item applies to your research, read the appropriate section before selecting a response.

Materials & experimental systems

n/a	Involved in the study	
☒	☐	Antibodies
☒	☐	Eukaryotic cell lines
☒	☐	Palaeontology and archaeology
☒	☐	Animals and other organisms
☒	☐	Human research participants
☒	☐	Clinical data
☒	☐	Dual use research of concern

Methods

n/a	Involved in the study	
☒	☐	ChIP-seq
☒	☐	Flow cytometry
☒	☐	MRI-based neuroimaging
